# Erlotinib in a once-daily high-dose oral regimen could mitigate Chikungunya virus titer in mice

**DOI:** 10.1371/journal.pone.0360169

**Published:** 2026-11-02

**Authors:** Chidinma Nelson Orji, Naphat Loeanurit, Akeanan Sroithongmoon, Noppadol Wacharachaisurapol, Yaowatree Jumroen, Wanna Eiamart, Supeecha Wittayalertpanya, Weerachai Chaijamorn, Tatta Sriboonruang, Jirapat Vamananda, Tanatorn Khotavivattana, Siwaporn Boonyasuppayakorn

**Affiliations:** 1 Center of Excellence in Applied Medical Virology, Department of Microbiology, Faculty of Medicine, Chulalongkorn University and King Chulalongkorn Memorial Hospital, Bangkok, Thailand; 2 Medical Science Program, Faculty of Medicine, Chulalongkorn University, Bangkok, Thailand; 3 Center of Excellence in Natural Products Chemistry, Department of Chemistry, Faculty of Science, Chulalongkorn University, Bangkok, Thailand; 4 Center of Excellence in Clinical Pharmacokinetics and Pharmacogenomics, Department of Pharmacology, Faculty of Medicine, Chulalongkorn University, Bangkok, Thailand; 5 Chula Pharmacokinetic Research Center, Faculty of Medicine, Chulalongkorn University, Bangkok, Thailand; 6 Department of Pharmacy Practice, Faculty of Pharmaceutical Sciences, Chulalongkorn University, Bangkok, Thailand; 7 Pharmacometrics for Health Sciences Research Group, Faculty of Pharmaceutical Sciences, Chulalongkorn University, Bangkok, Thailand; Federal University Oye-Ekiti, NIGERIA

## Abstract

Chikungunya virus (CHIKV) is a re-emerging arbovirus that causes debilitating inflammatory arthritis, for which no approved antiviral therapy currently exists. This study investigated the potential of repurposing erlotinib, a tyrosine kinase inhibitor, as an anti-CHIKV agent. Erlotinib demonstrated promising *in vitro* antiviral activity, with EC_50_ values in the low micromolar range and a favorable selectivity index. However, *in vivo* evaluation in C57BL/6 mice revealed pharmacokinetic limitations, characterized by rapid absorption and clearance, resulting in insufficient drug exposure under standard dosing regimens. Escalation to an intensive four-times-daily (QID) regimen successfully maintained plasma concentrations above the *in vitro* therapeutic threshold but led to significant body weight loss and failed to reduce viral titers or viral RNA levels in infected tissues. However, a once-daily (OD) regimen improved tolerability and significantly reduced footpad and serum viral titers relative to vehicle-treated controls. Notably, despite the lack of virological control in the QID regimen, erlotinib treatment reduced leukocyte infiltration and tissue inflammation in both treatment regimens, indicating a host-directed immunomodulatory effect. Overall, these findings highlight the critical role of dosing frequency in determining erlotinib's *in vivo* antiviral efficacy against CHIKV and suggest that once-daily (OD) administration may represent a more viable dosing strategy for further evaluation of erlotinib as a host-directed antiviral against CHIKV. Future strategies should also explore combination approaches with direct-acting antivirals to achieve more viral suppression and mitigation of disease pathology, as well as Structure-Activity Relationship (SAR)-guided scaffold optimization to improve *in vivo* pharmacokinetic profiles.

## Introduction

Chikungunya virus (CHIKV), a re-emerging alphavirus of the *Togaviridae* family, continues to pose a serious global health threat, leading to substantial morbidity and economic burden [1]. Transmitted by *Aedes aegypti* and *Aedes albopictus* mosquitoes, whose habitats are expanding due to climate change and urbanization, CHIKV infection causes an acute febrile illness characterized by maculo-papular rash and debilitating polyarthralgia [2,3]. The pathogenesis often involves chronic inflammation in multiple joints, which can persist for years and severely impact quality of life [4,5]. Despite recent advances in vaccine development, there is still no approved specific antiviral treatment, leaving patient care entirely dependent on supportive measures [6,7].

Currently, the management of both acute and chronic chikungunya disease is limited to symptomatic relief, primarily through nonsteroidal anti-inflammatory drugs (NSAIDs) and other palliative treatments [5]. This therapeutic gap highlights an urgent need for effective antiviral agents. Drug repurposing offers a promising strategy to accelerate the development of new therapies [8,9]. We noted that erlotinib, a compound previously reported as a potential treatment for rheumatoid arthritis could be a viable candidate for multiple viral infections, including respiratory syncytial virus, hepatitis C virus and Ebola virus, as well as the related mosquito-borne viruses such as dengue, Zika, and West Nile virus [10–12]. Therefore, we sought to explore whether this compound could serve as a potential therapeutic lead against the Chikungunya virus.

Erlotinib is an FDA approved type I tyrosine kinase inhibitor that reversibly binds to the catalytic domain of the epidermal growth factor receptor (EGFR) for the treatment of non-small cell lung cancer and pancreatic cancer [13]. Its potential as a broad-spectrum antiviral arises from its ability to modulate host cell processes exploited by many viruses. Erlotinib has been shown to impair intracellular viral trafficking by inhibiting host kinases AAK1 and GAK, which are crucial for clathrin-mediated endocytosis—a pathway used by CHIKV for cellular entry [11,14]. Furthermore, erlotinib can enhance the host's antiviral interferon response by decreasing STAT3 phosphorylation [15], making it a compelling candidate for repurposing as a host-targeted antiviral agent against the chikungunya virus.

## Materials and methods

### Compound

Erlotinib was purchased from MedChemExpress, New Jersey, USA. Ribavirin was obtained from Bio Basic in, Ontario, Canada.

### Cells and viruses

Vero (ATCC^®^CCL-81™), 293 (ATCC^®^CRL-1573™), RD (ATCC^®^CCL-136™), and C6/36 (ATCC^®^CRL-1660™) cell lines were cultured following established protocols [16]. The CHIKV (ECSA strain, accession number: MN114290) and a clinical CHIKV isolate (ECSA strain, accession number: PZ896987) were propagated in C6/36 cells using methods previously described in dengue virus [16].

### *In vitro* cytotoxicity and efficacy

The efficacy of erlotinib against CHIKV was assessed using the previously described methods [17]. Briefly, Vero cells (5 × 10^4^ cells/well) were seeded in 24-well plates and incubated overnight at 37 °C under a humidified chamber with 5% CO_2_. Following infection with CHIKV, the antiviral efficacy of erlotinib was evaluated by determining the 50% effective concentration (EC_50_) values using a plaque assay, as previously described [18]. In parallel, the 50% cytotoxic concentration (CC_50_) was assessed following established protocols [19]. Vero cells (1 × 10^4^ cells/well) were seeded in 96-well plates and incubated overnight before treatment with serial dilutions of erlotinib for 48 h. Cytotoxic effects were measured using the MTS assay (Promega, Madison, WI, USA), and the absorbance (*A*_*490*_) was measured using the Victor X3 Multi-Mode Microplate Reader (PerkinElmer, Shelton, CT, USA). The cytotoxicity was calculated as a percentage of the DMSO-treated control. EC_50_ and CC_50_ were determined by nonlinear regression in GraphPad Prism (GraphPad Software, San Diego, CA, USA). Data are presented as the mean ± standard error of the mean (SEM) from three independent experiments. The selectivity index (SI) was calculated as the ratio of CC_50_ to EC_50_. Similarly, CC50s were assessed in 293 and RD cells, and the EC50 of a clinical isolate was assessed in Vero cells. Moreover, an erlotinib antiviral inhibition was explored in both laboratory and clinical strains in HEK-293 and RD cells.

### Animal experiment

#### Mice.

Approval for the Animal Care and Use Protocol was granted by the Institutional Animal Care and Use Committee of the Faculty of Medicine, Chulalongkorn University, Bangkok, Thailand (project number: 2591005); in accordance with ARRIVE guidelines by the U.S National Institutes of Health for laboratory animal care and use. Seven-week–old adult C57BL/6 mice (18–22 g) were purchased from Nomura Siam International, Bangkok, Thailand. Mice were anesthetized with 4% isoflurane before daily blood collection and euthanized by cardiac puncture for an endpoint blood collection and cervical dislocation before harvesting footpad tissue.

#### Animal toxicity test.

For a single-dose oral toxicity study, twelve C57BL/6 mice were evenly divided into two groups: one receiving erlotinib treatment and the other serving as the vehicle control. Erlotinib was formulated in the vehicle (consisting of 0.3% w/v of sodium carboxymethylcellulose and 0.1% v/v of Tween 80 in water) prior to administration [20]. The treatment group (n = 6) received erlotinib at 30 mg/kg via oral administration, while the control group (n = 6) was given only the vehicle. Clinical signs were monitored daily, and blood samples were collected on days 1, 3, and 7 post administration to analyze alanine transaminase (ALT) (Abcam Mouse ALT ELISA Kit; Cambridge, UK), and serum creatinine (Cr) (QuantiChromTM, DICT-500, BioAssay, Hayward, CA, USA). In addition, one mouse was included in each group to obtain liver and kidney samples for histopathological examination using H & E staining.

To evaluate the repeated dose regimen, mice were administered 30 mg/kg erlotinib orally once daily for three consecutive days. On day 4, blood samples were taken to assess ALT and Cr levels as described above. Clinical signs were monitored daily, and liver and kidney tissues were taken for histopathological examination using H & E staining.

To evaluate higher exposure levels and alternative administration routes, the study was further extended to assess multiple-dose toxicity with escalated dosing. Twenty-four mice were divided equally into four groups: 100 mg/kg intraperitoneal (IP), 100 mg/kg oral, 150 mg/kg oral, and vehicle control. Treatments were given once daily for three consecutive days. Clinical signs were tracked daily, and blood samples were collected on day 4 after administration for ALT and Cr analysis. One mouse per group was sacrificed for histopathological examination of liver and kidney tissues.

Finally, given the rapid absorption of erlotinib observed following once-daily dosing, intensive multiple-dose toxicity was evaluated using higher-frequency administration. Twelve mice were divided equally into two groups and received oral erlotinib at either 100 mg/kg or 150 mg/kg, administered four times daily (QID) for three consecutive days. Clinical signs were observed daily, and blood samples were obtained on day 4 post-administration. Plasma was analyzed for ALT and Cr as previously described. Additionally, one mouse from each group was sacrificed to obtain liver and kidney tissues for histopathological analysis.

#### Pharmacokinetic study.

*Sample preparation:* To measure the levels of erlotinib in mice, plasma samples were processed using the acetonitrile (ACN) protein precipitation technique. Briefly, 10 µL of plasma was mixed with 70 µL of acetonitrile, followed by vigorous vortexing for approximately 1 minute. The mixture was centrifuged for 5 minutes, and the resulting supernatant was transferred into vials. Samples were kept in the autosampler at 15 °C, and a 5 µL aliquot was injected into the LC–MS/MS system for analysis.

*LC-MS/MS condition:* Erlotinib was quantified using an LC-MS/MS (Shimadzu Corporation, Kyoto, Japan) equipped with a Column Oven (CTO-20A), Detector (LCMS-8040), Autosampler (SIL-20ACXR), Pump (LC-20ADXR), Degasser unit (DGU-20A3R), and Valve unit (FCV-20AH2), operated with Lab solutions software version 6.90 SP2. Chromatographic separation was achieved using an Avantor ACE C18 column (100 × 3 mm, 3 µm particle size) coupled with a Phenomenex C18 guard column (4.0 × 2.0 mm). The autosampler was set at 15 °C while the column oven temperature was set at 40 °C. The gradient mobile phase, comprising 0.1% (v/v) formic acid in water and acetonitrile, was delivered at a flow rate of 0.4 mL/min over a 5.5-minute run time. Multiple reaction monitoring (MRM) of the transition m/z 393.9 → 278.0 was employed in positive ion mode (S1A Fig).

*Validation of analytical method:* The specificity of the method was assessed by comparing blank mouse plasma with erlotinib-spiked plasma samples during each analytical run. Interference at the retention time of the analyte was evaluated prior to study sample analysis. The method demonstrated high selectivity for erlotinib, with no significant interference from endogenous plasma components at the corresponding retention time. Linearity of erlotinib quantification was assessed using calibration curves created from eight concentration levels ranging from 0.07 to 3,500 ng/mL (S1B Fig and S1A Table). To plot the calibration curve, linear least-squares regression with a weighting factor of 1/C^2^ was applied, plotting the peak area of test standards against nominal concentrations. The back-calculated concentrations of the analytes ranged from 89.7% to 109.6%, which falls within the accepted ±15% range. The coefficient of determination (R^2^) exceeded 0.99, confirming that the method was valid, linear, and accurate for erlotinib quantification.

*Pharmacokinetic experiment and data analysis:* To investigate the pharmacokinetic profile of erlotinib in mice, pharmacokinetic (PK) parameters were calculated using non-compartmental analysis (NCA) based on sparse serial blood sampling across alternative groups of animals (S1B Table). This strategy was chosen to minimize the risk of hypovolemic shock, which can occur when excessive blood is withdrawn from the same mouse beyond the acceptable volume range [21,22]. Eight-week-old mice (n = 24; 4 alternating groups × 6 mice) were orally administered 200 µL of erlotinib (30 mg/kg) prepared in vehicle (0.3% w/v sodium carboxymethylcellulose and 0.1% v/v Tween 80 in water). Blood samples were obtained via tail vein nicking at predetermined time points (0.5, 1, 2, 3, 4, 5, 6, 12, and 24 h) after dosing. The plasma samples were stored at –80 °C. PK parameters were calculated using Phoenix™ 64 software (version 8.3, Certara, Princeton, NJ) based on an NCA approach, as previously described [18]. Parameters reported included area under the plasma concentration–time curve from 0 to t (AUC_0–t_), time to C_max_ (T_max_), maximum plasma concentration (C_max_), elimination half-life (t_1/2_), volume of distribution (V_d_), area under the curve extrapolated to infinity (AUC_0–inf_), and clearance (CL).

Following the full single-dose PK profile of erlotinib at 30 mg/kg, the pharmacokinetics of erlotinib were further evaluated at escalated doses of 100 mg/kg and 150 mg/kg administered under a multiple-dose regimen. C57BL/6 mice were assigned randomly into groups (n = 6 per dose group) and received erlotinib orally by gavage once daily for three consecutive days. Dosing solutions were prepared in vehicle (0.3% w/v sodium carboxymethylcellulose and 0.1% v/v Tween 80 in water). The blood samples were obtained via tail vein nicking at defined time points relative to dosing. Specifically, samples were obtained 1 h prior to dosing (−1 h) to establish baseline concentrations and 1 h post-dosing (+1 h) to capture peak absorption on days 0, 1, and 2 after compound administration. These time points were selected to provide insight into early absorption, short-term elimination dynamics, and potential drug accumulation across dosing intervals. Plasma was separated by centrifugation and stored at −80 °C. Plasma concentrations of erlotinib were quantified using LC–MS/MS as described above. Non-compartmental analysis was performed in Phoenix™ (version 8.3, Certara, Princeton, NJ) to determine pharmacokinetic parameters, including C_max_, T_max_, AUC, and t_1/2_.

Finally, to evaluate drug exposure under a repeated dosing regimen and assess potential accumulation, erlotinib was administered orally to C57BL/6 mice at doses of 100 mg/kg or 150 mg/kg (n = 6 per group). The compound was given four times daily (QID., every 6 hours) for three consecutive days. Blood samples were collected once daily, immediately before the first administration of each new day, in order to assess systemic drug levels resulting from the previous day’s dosing. This design allowed evaluation of potential drug accumulation across the dosing period. Plasma was separated by centrifugation and stored at −80 °C. Plasma concentrations of erlotinib were quantified using LC–MS/MS as described above.

#### *In vivo* anti-CHIKV efficacy experiment.

Two independent *in vivo* efficacy studies were conducted in C57BL/6 mice to evaluate the antiviral and immunomodulatory effects of erlotinib using distinct dosing regimens: an intensive four-times-daily regimen (QID) and a once-daily regimen (OD). Each experiment was performed using separate cohorts of mice.

For both studies, mice were randomly assigned to experimental groups including: (i) mock-infected/uninfected control, (ii) CHIKV-infected untreated control, (iii) infected vehicle-treated, (iv) infected erlotinib-treated, and (v) infected ribavirin-treated drug control. The mock-infected group was inoculated subcutaneously (s.c.) in the left hind footpad with MEM, whereas all other groups were inoculated s.c. with CHIKV (5 × 10⁶ PFU/mouse) in MEM.

In the QID efficacy study, treatment was initiated at 24 h post-infection. Erlotinib was administered orally at 100 mg/kg four times daily (every 6 h) for three consecutive days. The vehicle-treated group received the formulation buffer (0.3% w/v sodium carboxymethylcellulose and 0.1% v/v Tween 80 in water) following the same dosing schedule. Ribavirin was administered intraperitoneally (IP) at 50 mg/kg twice daily (BID) as a positive control. We used ribavirin as a positive control. We are aware that ribavirin is not an ideal positive control as anti-CHIKV antiviral due to its varied efficacy across systems [23,24]. However, considering there is no approved anti-CHIKV drug available and ribavirin is a broad-spectrum nucleoside analog that works through multiple direct and indirect mechanisms to disrupt viral replication [25], while also being occasionally selected as a symptomatic treatment in clinical settings [26,27], it served as an acceptable benchmark for our *in vivo* efficacy assays.

In a separate experiment, the OD regimen was evaluated using an independent cohort of mice. Erlotinib was administered orally at 100 mg/kg once daily (OD) for three consecutive days, beginning at 24 h post-infection. Vehicle-treated controls received the same formulation buffer following the OD schedule. No direct comparisons between QID and OD groups were performed, as the experiments were conducted independently.

In both studies, mice were monitored daily for clinical signs of disease, including body weight, physical activity, and local inflammation at the site of infection. Footpad swelling was assessed by measuring both width and length using a digital caliper. Activity was scored based on spontaneous movement and feeding behavior using a previously described [28,29] scale ranging from 1 (very active) to 5 (lethargic).

To assess antiviral efficacy, blood and footpad tissue samples were collected at designated time points (n = 6 per group per time point). Serum and tissue homogenates were prepared as previously described. Viral titers were quantified using plaque assays and expressed as PFU/mL (serum) or PFU/g (tissue), while viral RNA levels were determined by RT-qPCR and reported as copies/mL [18].

To evaluate host inflammatory responses, cytokine expression levels in both footpad tissues and blood samples were analyzed by quantitative RT-PCR. Gene expression was normalized to housekeeping genes (β-actin and HPRT), and relative expression levels were calculated using the ΔΔCt method. All primer sequences are listed in the S2 Table. Additionally, footpad tissues were collected for histopathological analysis. Samples were fixed, sectioned, and stained with hematoxylin and eosin (H&E) to assess leukocyte infiltration, edema, and tissue architecture. Histological tissue inflammation was scored, and statistical analysis was performed using two-way ANOVA with Dunnett’s post-hoc test against vehicle controls with significance set at * p < 0.05. The histological scoring system followed the previous description [18,30]. Relative gene expression (${\text{2}}^{-\Delta \Delta C_{t}}$) for cytokines and footpad viral RNA, viral titers (footpad and serum), and serum viral RNA copies were log-transformed (${\text{log}}_{\text{2}}$ or ${\text{log}}_{\text{1}\text{0}}$) to satisfy parametric assumptions of normality and equal variance. Group and time-point differences were evaluated using two-way ANOVA with Dunnett’s post-hoc test against vehicle controls [11] with statistical significance set at *p<0.05. Values below the assay's limit of detection (LOD) were assigned half the LOD prior to log10 transformation and statistical analysis.

All samples were processed immediately after collection and stored at −80 °C until analysis. Statistical analysis of clinical parameters (body weight, activity scores, and footpad dimensions) was performed using unpaired Student’s t-test, with p < 0.05 considered statistically significant.

## Results

### *In vitro* cytotoxicity and efficacy of erlotinib

Erlotinib, a U.S. FDA–approved tyrosine kinase inhibitor, was selected as a candidate for drug repurposing based on high-throughput screening results [31] and prior evidence of activity against other RNA viruses. We first evaluated its antiviral efficacy against CHIKV (Fig 1). In Vero cells, erlotinib inhibited replication of both the laboratory-adapted strain and a clinical isolate, with EC_50_ values of 3.79 ± 0.89 µM (SI ≈ 15.85) and 4.97 ± 2.02 µM (SI ≈ 12.07), respectively (Fig 1A–B). These values were determined in the context of a CC₅₀ of approximately 60 µM in Vero cells (Fig 1C).

**Fig 1:**
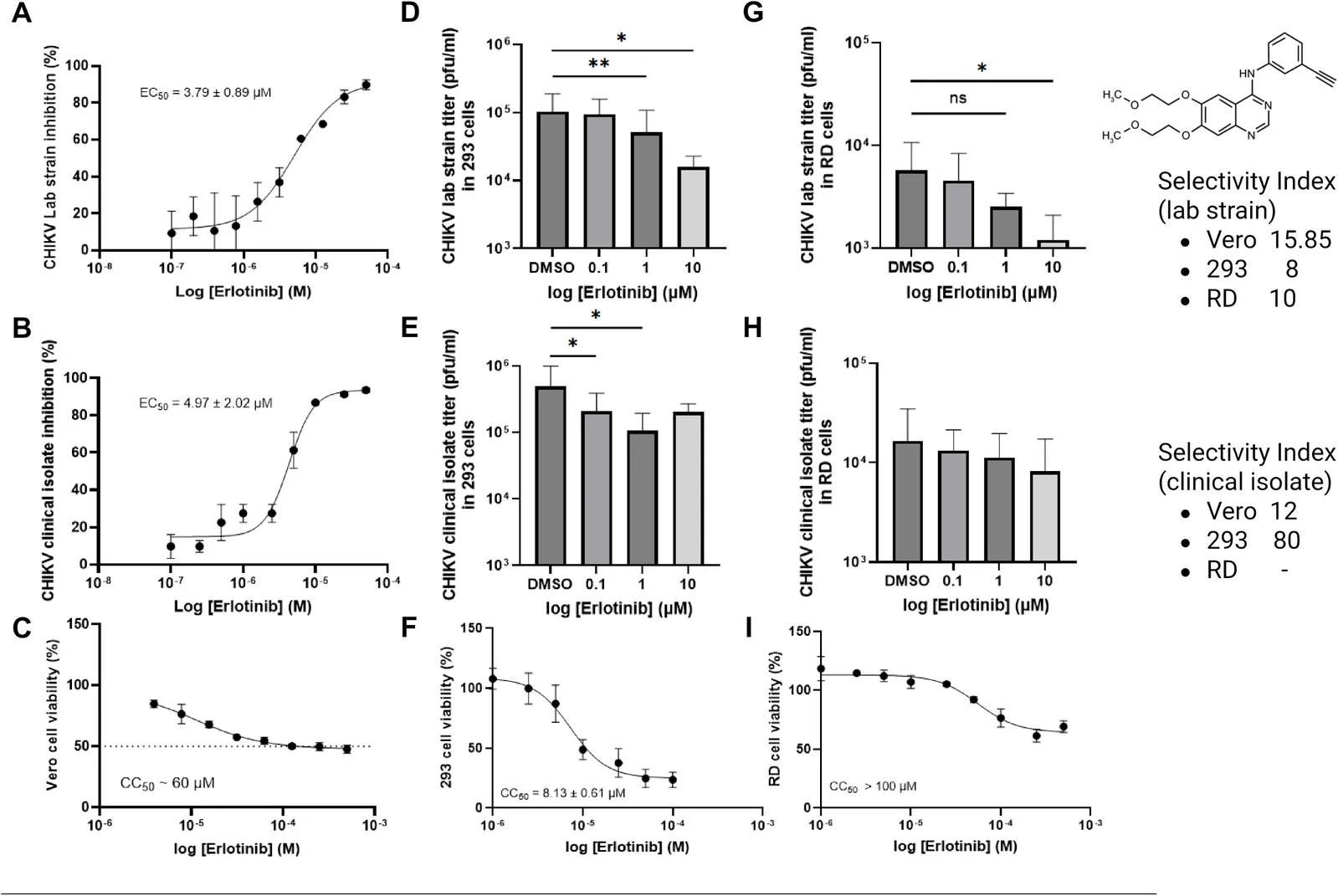
Antiviral activity and cytotoxicity of erlotinib against CHIKV in multiple cell lines. (A–B) Dose–response curves showing the inhibitory effect of erlotinib on Chikungunya virus (CHIKV) replication in Vero cells infected with (A) laboratory-adapted strain and (B) clinical isolate. Viral titers were quantified by plaque assay and expressed as percent inhibition. The half-maximal effective concentration (EC_50_) values were determined by nonlinear regression (log (inhibitor) vs response – variable slope (four parameter)) in GraphPad Prism. (C) Cytotoxicity of erlotinib in Vero cells, measured by cell viability assay after 48 h treatment. The half-maximal cytotoxic concentration (CC_50_) was determined by nonlinear regression (log (inhibitor) vs response – variable slope (four parameter)) in GraphPad Prism. (D–E) Dose-dependent inhibition of CHIKV replication in 293 cells infected with (D) laboratory strain and (E) clinical isolate following treatment with increasing concentrations of erlotinib (0.1–10 µM). Viral titers were determined by plaque assay. Statistical significance was analyzed relative to DMSO-treated controls (*p < 0.05, **p < 0.01, paired t-test). (F) Cytotoxicity profile of erlotinib in 293 cells, with CC₅₀ determined by nonlinear regression (log (inhibitor) vs response – variable slope (four parameter)) in GraphPad Prism. (G–H) Antiviral activity of erlotinib in RD cells infected with (G) laboratory strain and (H) clinical isolate, showing reduced viral titers upon treatment. Statistical significance is indicated (*p < 0.05; ns, not significant, paired t-test). (I) Cytotoxicity of erlotinib in RD cells, demonstrating minimal toxicity at tested concentrations (CC_50_ > 100 µM). Selectivity index (SI), calculated as CC_50_/EC_50_, is summarized for each cell type and viral strain. Data represents SD from at least three independent experiments.

We next assessed antiviral activity and cytotoxicity in human-derived cell lines, HEK293 and RD cells (Fig 1D–I). HEK293 cells (human embryonic kidney origin) exhibited dose-dependent inhibition, with ~50% reduction in viral titers observed at 1 µM and 0.1 µM for the laboratory strain and clinical isolate, respectively (Fig 1D–E). However, the CC₅₀ in HEK293 cells was 8.13 ± 0.61 µM (Fig 1F), indicating a relatively narrow therapeutic window and suggesting that inhibition observed at 10 µM may be partially confounded by cytotoxic effects. The corresponding selectivity indices were estimated to be ~ 8 for the laboratory strain and ~80 for the clinical isolate.

In RD cells (human rhabdomyosarcoma origin), erlotinib significantly reduced replication of the laboratory strain only at 10 µM (Fig 1G), while cytotoxicity remained low (CC_50_ > 100 µM; Fig 1I), yielding an estimated SI of ~10. In contrast, the clinical isolate exhibited no significant response to treatment in RD cells (Fig 1H), precluding SI determination.

Collectively, these findings demonstrate that erlotinib exerts cell type– and isolate-dependent antiviral activity against CHIKV, supporting its potential as a lead compound for further anti-CHIKV development, while highlighting the importance of therapeutic window considerations in human cells.

### Toxicity and pharmacokinetic (PK) evaluation of erlotinib (30 mg/kg) in C57BL/6 mice.

The safety profile of erlotinib evaluated in C57BL/6 mice was formulated in a vehicle (consisting of 0.3% w/v of sodium carboxymethylcellulose and 0.1% v/v of Tween 80 in water) before administration [20]. The 30 mg/kg dose was selected because it was the mouse equivalent of the human clinical dose for non-small cell lung cancer (NSCLC) (18). The objective of this experiment is to evaluate whether the 30 mg/kg dose would elicit a desirable drug level in the blood without causing toxicity.

First, an acute single-dose study was performed (Fig 2A). Following a single oral 30 mg/kg dose, mice showed no significant changes in daily clinical activity scores (Fig 2B) or body weight (Fig 2C) compared to vehicle controls over 7 days post-administration. Furthermore, serum levels of the liver and kidney biomarkers ALT (Fig 2D) and creatinine (Fig 2E) remained within normal ranges. Histopathological analysis of the kidney, liver, and spleen on days 1, 3, and 7 revealed no treatment-related abnormalities (S2 Fig).

**Fig 2:**
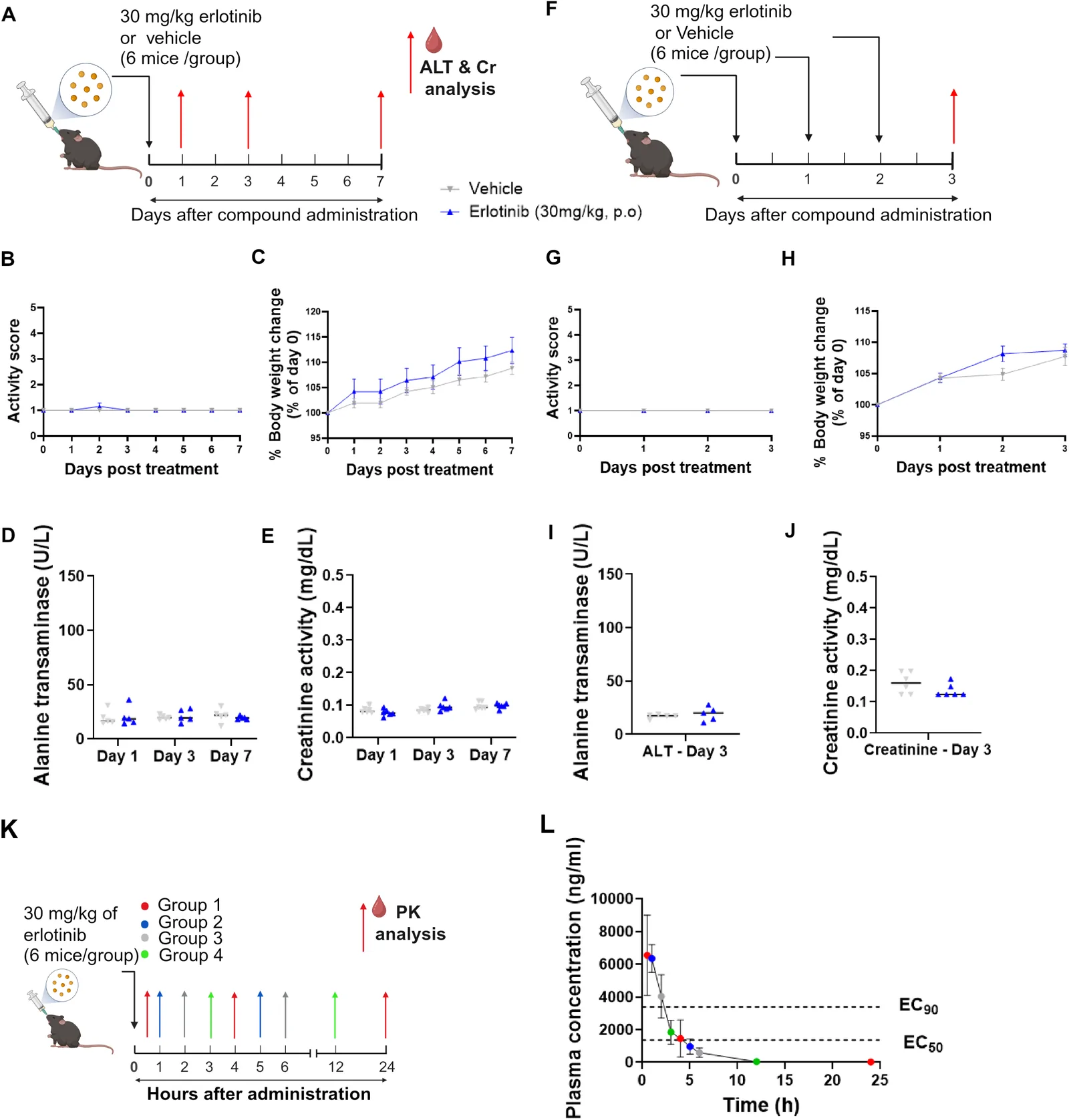
Toxicity and pharmacokinetic (PK) evaluation of erlotinib (30 mg/kg) in C57BL/6 mice. (A) Schematic of the single-dose toxicity study (30 mg/kg) created with BioRender.com. (B) Daily activity scores and (C) percent body weight change over 7 days. (D) Serum ALT and (E) creatinine levels on days 1, 3, and 7. (F) Schematic of the multiple-dose toxicity study (30 mg/kg, once daily, for 3 days) created with BioRender.com. (G) Daily activity scores and (H) percent body weight change during treatment. (I) Serum ALT and (J) creatinine levels on day 3. (K) Schematic of the single-dose PK study (30 mg/kg) created with BioRender.com. (L) Plasma concentration-time profile after a single 30 mg/kg dose. Dotted lines represent *in vitro* EC_50_ and EC_90_ levels corresponding to the plasma concentration.

Next, a multiple-dose study was conducted (Fig 2F), with mice receiving 30 mg/kg erlotinib orally once daily for three consecutive days. Similar to the single-dose study, this regimen did not impact clinical activity scores (Fig 2G) or body weight (Fig 2H). On day 3, serum ALT (Fig 2I) and creatinine (Fig 2J) levels were normal. Histology of the kidney, liver, and spleen was indistinguishable from vehicle-treated controls, showing no evidence of inflammation, tissue damage, or lymphoid depletion (S3 Fig).

To evaluate the plasma-concentration profile, a single 30 mg/kg oral dose of erlotinib was administered to C57BL/6 mice, as outlined in the schematic (Fig 2K). Briefly, all 24 mice were administered simultaneously and were divided into 4 groups to alternatively withdraw the blood at respective time-points to minimize the risk of hypovolemic shock [32]. The resulting plasma concentration-time profile (Fig 2L) shows that erlotinib was rapidly absorbed, achieving a peak concentration (C_max_) significantly above both the *in vitro* EC_50_ and EC_90_ levels. However, the drug was also removed rapidly from the circulation. The plasma concentration dropped below the EC_90_ threshold at approximately 2 hours post-administration and fell below the EC_50_ threshold by the 4-hour mark. This profile indicates a rapid absorption followed by a relatively short-lived systemic exposure above the target *in vitro* effective concentrations.

The detailed PK profile of erlotinib was performed in mice using a validated LC–MS/MS method. It was successfully detected without significant interference, with a retention time of 2.9 min (S4 Fig). Calibration standards ranged from 0.070 to 3,500 ng/mL, yielding a regression equation of Y = 15,293.6X + 2,554.81 (S1B Fig). Back-calculated concentrations were 89.70–109.65%, with all calibration curves showing *R*^*2*^ > 0.99 (S1A Table), confirming the accuracy and linearity of the method. Pharmacokinetic evaluation was then conducted to assess the absorption and distribution in mice. Plasma concentration–time profiles were generated from serial blood sampling at different time points (Table 1). Erlotinib reached a peak plasma concentration (C_max_) of 6547.44 ng/mL at 0.5 h (T_max_), indicating rapid absorption. The elimination half-life (t_1/2_) was 2.72 h, consistent with a short systemic residence time. The total systemic exposure, expressed as area under the curve (AUC), was 17,923.1 ng·h/mL. These findings suggest that while erlotinib is rapidly absorbed in mice, its circulation time is short, resulting in limited exposure above therapeutic levels.

**Table 1:** Pharmacokinetic parameters of erlotinib following oral administration at 30 mg/kg in mice.

Parameter	Mean value (n = 6 mice)
HL (h)	2.72
Tmax (h)	0.5
Cmax (ng/mL)	6547.44
AUClast (h*ng/mL)	17923.08
AUCINF_obs (h*ng/mL)	18002.13
AUC_%Extrap_obs (%)	0.44
Vz_F_obs (mL/kg)	6542.25
Cl_F_obs (mL/h/kg)	1666.47
MRTlast (h)	2.69
MRTINF_obs (h)	2.8

In summary, erlotinib at 30 mg/kg was well tolerated as both a single dose and a three-day, once-daily regimen, showing no acute toxicity. However, the PK profile, while showing rapid absorption, indicated that systemic exposure above the *in vitro* effective concentrations was transient.

### High-dose toxicity and pharmacokinetic (PK) evaluation of erlotinib

Given the transient plasma concentrations observed at 30 mg/kg, we next evaluated higher dosing regimens to determine if sustained therapeutic exposure could be achieved without inducing toxicity. A multiple-dose study was conducted where mice received erlotinib once daily (OD) for three days at 100 mg/kg (intraperitoneal, IP), 100 mg/kg (per oral, PO), or 150 mg/kg (PO), compared to a vehicle control (Fig 3A).

**Fig 3:**
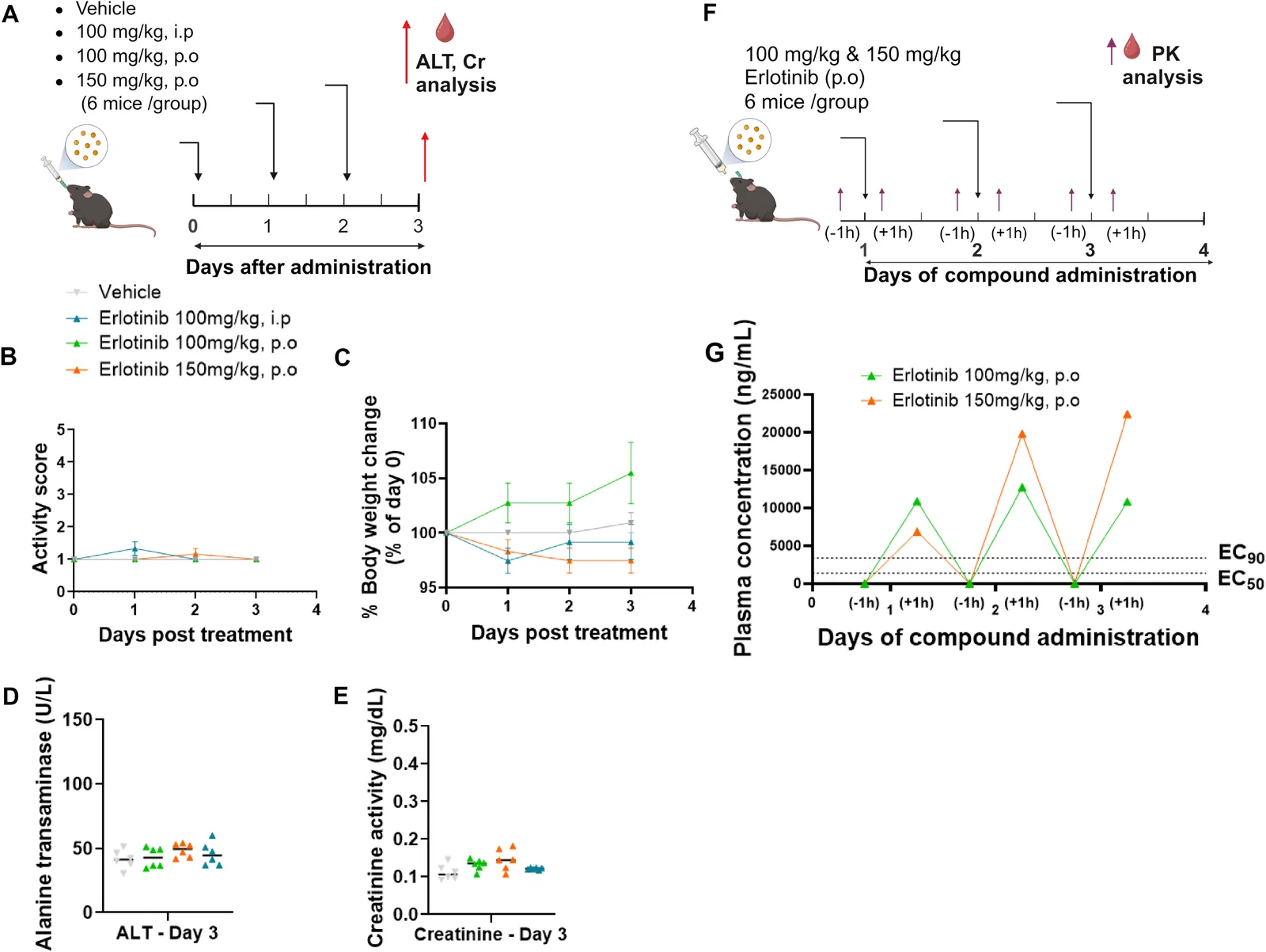
Toxicity and plasma concentration level evaluation of high-dose erlotinib in C57BL/6 mice. (A) Schematic of the multiple-dose toxicity study, where mice (n = 6 per group) received vehicle, 100 mg/kg erlotinib (IP), 100 mg/kg erlotinib (PO), or 150 mg/kg erlotinib (PO), once daily for 3 days created with BioRender.com. (B) Daily activity scores and (C) percent body weight change during the 3-day treatment. (D) Serum ALT and (E) creatinine levels measured on day 3. (F) Schematic of the multiple-dose PK experiment (100 mg/kg or 150 mg/kg, PO) with measurements at 1 h pre-dose and 1 h post-dose for 3 days created with BioRender.com. (G) Plasma concentrations of erlotinib measured 1 h pre-dose and 1 h post-dose. Data are presented as mean ± SEM.

All high-dose regimens were well tolerated. No significant changes were observed in daily activity scores (Fig 3B) or body weight (Fig 3C) between treatment and vehicle groups. Furthermore, serum levels of ALT (Fig 3D) and creatinine (Fig 3E) on day 3 were normal and indistinguishable from controls, indicating that these doses did not cause detectable acute hepatic or renal toxicity. Histopathological analysis showed no treatment-related abnormalities in the kidney and spleen; however, mild vacuolization of hepatocytes was observed in the liver, suggesting transient drug-induced hepatitis (S5 Fig).

The multiple-dose PK profile of the 100 mg/kg and 150 mg/kg oral regimens was assessed by measuring plasma concentrations 1 hour before (trough) and 1 hour after (peak) drug administration (Fig 3F). The pharmacokinetic analysis revealed a dose-dependent, non-linear increase in exposure (Table 2). While the half-life (HL) (approx. 2 h) and time to maximum concentration (T_max_) (1 h) were similar for both doses, increasing the dose 1.5-fold (from 100 to 150 mg/kg) resulted in a 1.79-fold increase in C_max_ (11,799 vs. 21,126 ng/mL) and a 1.9-fold increase in total exposure (AUClast: 39,494 vs. 75,063 ng·h/mL). Also, although peak concentrations (+1 h) for both regimens were high and rose well above the *in vitro* EC_90_ threshold, the trough concentrations (−1 h) on days 2 and 3 dropped to near-undetectable levels (Fig 3G). This indicates rapid clearance, consistent with the short half-life, with no evidence of drug accumulation at 100 mg/kg and only minimal accumulation at 150 mg/kg.

**Table 2:** Pharmacokinetic parameters of erlotinib in mice following oral administration at 100 mg/kg or 150 mg/kg, measured at 1 h pre-dose and 1 h post-dose.

Parameter	100 mg/kg	150 mg/kg
HL (h)	1.98 ± 0.13	2.10 ± 0.57
Tmax (h)	1	1
Cmax (ng/mL)	11798.71 ± 2513.59	21126.49 ± 2949.47
AUClast (h*ng/mL)	39494.48 ± 8056.06	75062.64 ± 22334.62
AUCINF_obs (h*ng/mL)	39506.60 ± 8059.06	75217.31 ± 22619.54
AUC_%Extrap_obs (%)	0.03 ± 0.02	0.14 ± 0.27
Vz_F_obs (mL/kg)	7572.78 ± 1842.88	6167.36 ± 868.81
Cl_F_obs (mL/h/kg)	2642.01 ± 589.48	2150.00 ± 572.95

These results demonstrate that while high oral doses of 100 mg/kg and 150 mg/kg erlotinib are well tolerated, the drug is cleared too rapidly to maintain therapeutic trough plasma concentrations.

### Evaluation of an intensive QID dosing regimen

#### Toxicity and plasma drug level of QID dosing in non-infected mice.

Given the transient systemic exposure from once-daily dosing (Figs 2L and 3G; Tables 1, 2), an intensive regimen was evaluated to determine if therapeutic plasma concentrations could be sustained. A 6-hour dosing interval (four times daily, QID) was selected.

The safety of this QID regimen was first assessed in non-infected mice for three days (Fig 4A). Both the 100 and 150 mg/kg groups were generally well tolerated, with all animals surviving. Treated mice exhibited mild, non-significant reductions in physical activity (Fig 4B) but experienced a significant, dose-dependent body weight decrease of up to 15% by day 3 (Fig 4C). Importantly, no overt toxicity was detected via serum biomarkers, as both ALT (Fig 4D) and creatinine (Fig 4E) levels remained normal. Histopathological analysis confirmed this, showing the 100 mg/kg dose was tolerated, while the 150 mg/kg dose induced obvious pathological changes (e.g., slight tubular dilation, periportal hepatocyte granularity, lymphoid depletion) (S6 Fig).

**Fig 4:**
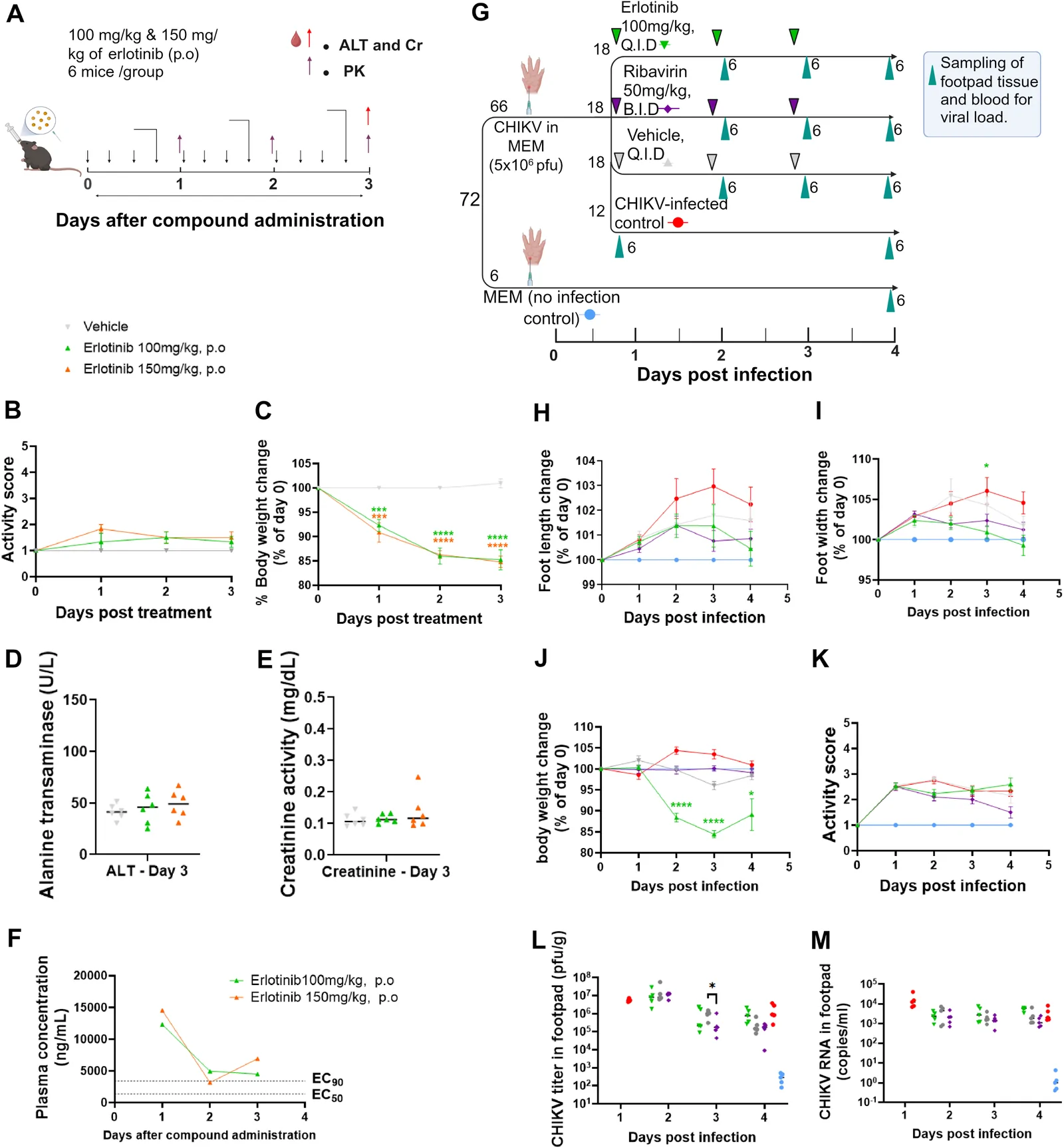
High-dose (QID) toxicity, plasma concentration level, and *in vivo* efficacy of erlotinib. (A-F) Toxicity and pharmacokinetic evaluation in non-infected C57BL/6 mice. (A) Schematic of the multiple-dose (QID) toxicity study (100 or 150 mg/kg, PO, for 3 days) created with Biorender.com. (B) Daily activity scores. (C) Percent body weight change. (D) Serum ALT and (E) creatinine levels on day 3. (F) Plasma concentration-time profile of erlotinib during the multiple-dose QID regimen, with *in vitro* EC_50_ and EC_90_ levels indicated. (G-M) *In vivo* efficacy evaluation in CHIKV-infected C57BL/6 mice. (G) Schematic of the *in vivo* efficacy study, with treatment (100 mg/kg, PO, QID) for 3 days post-infection created with Biorender.com. (H) Footpad length change (%). (I) Footpad width change (%). (J) Percent body weight change. (K) Clinical activity scores. (L) CHIKV titer in footpad (PFU/g). (M) CHIKV RNA in footpad (copies/mL). Data are presented as mean ± SEM (n = 5–6 per group). Statistical analysis of viral load was determined by two-way ANOVA on log-transformed values followed by Dunnett’s multiple comparisons test comparing treatments to vehicle control at each time point (p<0.05). Raw fold change values / viral titers are displayed on the Y-axis for visualization. Statistical analysis of clinical parameters was evaluated with unpaired t-test (*p* < 0.05).

Plasma concentration analysis confirmed that this intensive dosing strategy was successful (Fig 4F). This regimen sustained plasma trough concentrations for both the 100 and 150 mg/kg groups consistently above the *in vitro* EC_90_ threshold throughout the 3-day treatment period. Interestingly, the plasma concentrations in the 100 mg/kg group appeared more stable than those in the 150 mg/kg group. Therefore, the 100 mg/kg QID regimen was prioritized for the *in vivo* efficacy evaluation.

#### *In vivo* efficacy of QID high-dose erlotinib in CHIKV-infected mice.

Having established a tolerated regimen that maintained therapeutic plasma concentrations, we assessed its *in vivo* efficacy in a C57BL/6 mouse model of CHIKV infection (Fig 4G). Infected mice were treated with erlotinib (100 mg/kg, PO, QID) or a positive control, ribavirin (50 mg/kg, IP, BID), for three days. By 24 hours post-infection, CHIKV-infected mice developed mild foot swelling (Fig 4H-I), body weight changes (Fig 4J), and increased activity scores (Fig 4K), with footpad viral loads established at approximately 10^7^ PFU/g (Fig 4L) and 10^4^ copies/mL (Fig 4M), measured by plaque titration and RT-PCR, respectively.

From 48–96 hours post-infection, the 100 mg/kg QID treatment (400 mg/kg/day) provided no significant improvement in footpad length change (Fig 4H) or clinical activity scores (Fig 4K) when compared to the vehicle control on the respective date. While a significant reduction in footpad width was noted by 72 hours post-infection in erlotinib-treated mice (Fig 4I), these mice also experienced pronounced body weight loss (Fig 4J), consistent with the drug-associated toxicity observed in non-infected mice (Fig 4C), although recovery was observed by 96 hours. Crucially, erlotinib treatment failed to control viral replication. Tissue viral loads, measured by both plaque assay (Fig 4L) and RT-qPCR (Fig 4M), were not significantly different from the vehicle-treated group. In contrast, the ribavirin control significantly reduced viral titers at 72 hours post-infection (Fig 4L). However, despite its lack of antiviral activity, erlotinib mitigated acute inflammation. Histopathological analysis revealed that tissue inflammation was decreased in the erlotinib-treated group beginning at 48 hours post-infection, relatively similar to that of ribavirin treatment (Fig 5). The histopathological score of representative footpad sections also revealed a significant reduction in inflammation score in erlotinib-treated mice compared with vehicle-treated controls.

**Fig 5:**
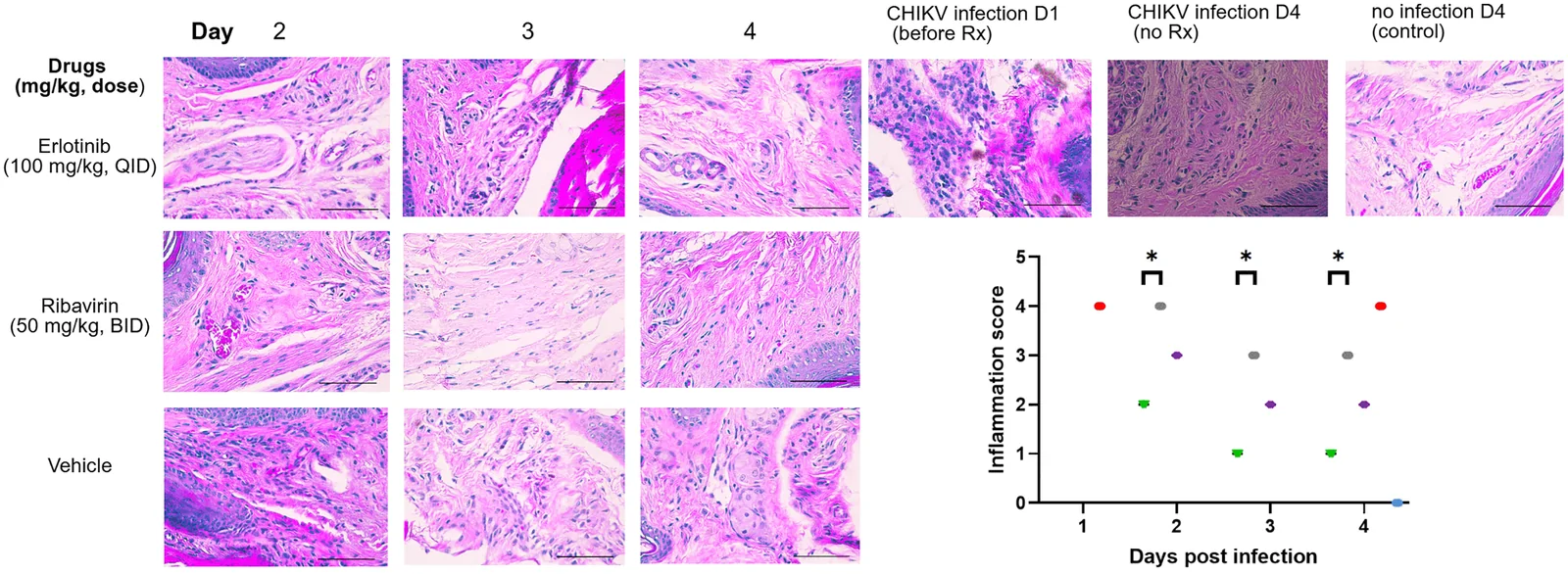
Histopathological analysis of footpad tissues of QID high-dose erlotinib in CHIKV-infected mice. Representative H&E-stained sections of footpad tissues. Controls include uninfected mice (Day 4) and CHIKV-infected mice prior to treatment (Day 1 post-infection). Treatment groups show tissue collected after 1, 2, and 3 days of administration with vehicle, erlotinib (100 mg/kg, QID), or ribavirin (50 mg/kg, IP, BID). Sections were assessed for leukocyte infiltration and structural changes. The scale bars represented 100 µm. Histological tissue inflammation was scored, and statistical analysis was performed using two-way ANOVA followed by Dunnett’s multiple comparisons test comparing treatments to vehicle control at each time point with significance set at * p < 0.05.

Given that the QID regimen is not clinically practical and was associated with body weight loss, we next investigated whether a simplified once-daily (OD) dosing strategy could retain therapeutic benefit while improving tolerability.

### *In vivo* efficacy of OD high-dose erlotinib in CHIKV-infected mice

To further evaluate the *in vivo* activity of erlotinib under a less intensive and clinically relevant dosing regimen, we assessed its efficacy at 100 mg/kg administered once daily (OD) in CHIKV-infected C57BL/6 mice (Fig 6A). Treatment was initiated at 24 h post-infection, clinical parameters and antiviral activity were evaluated by measuring viral loads and host responses.

**Fig 6:**
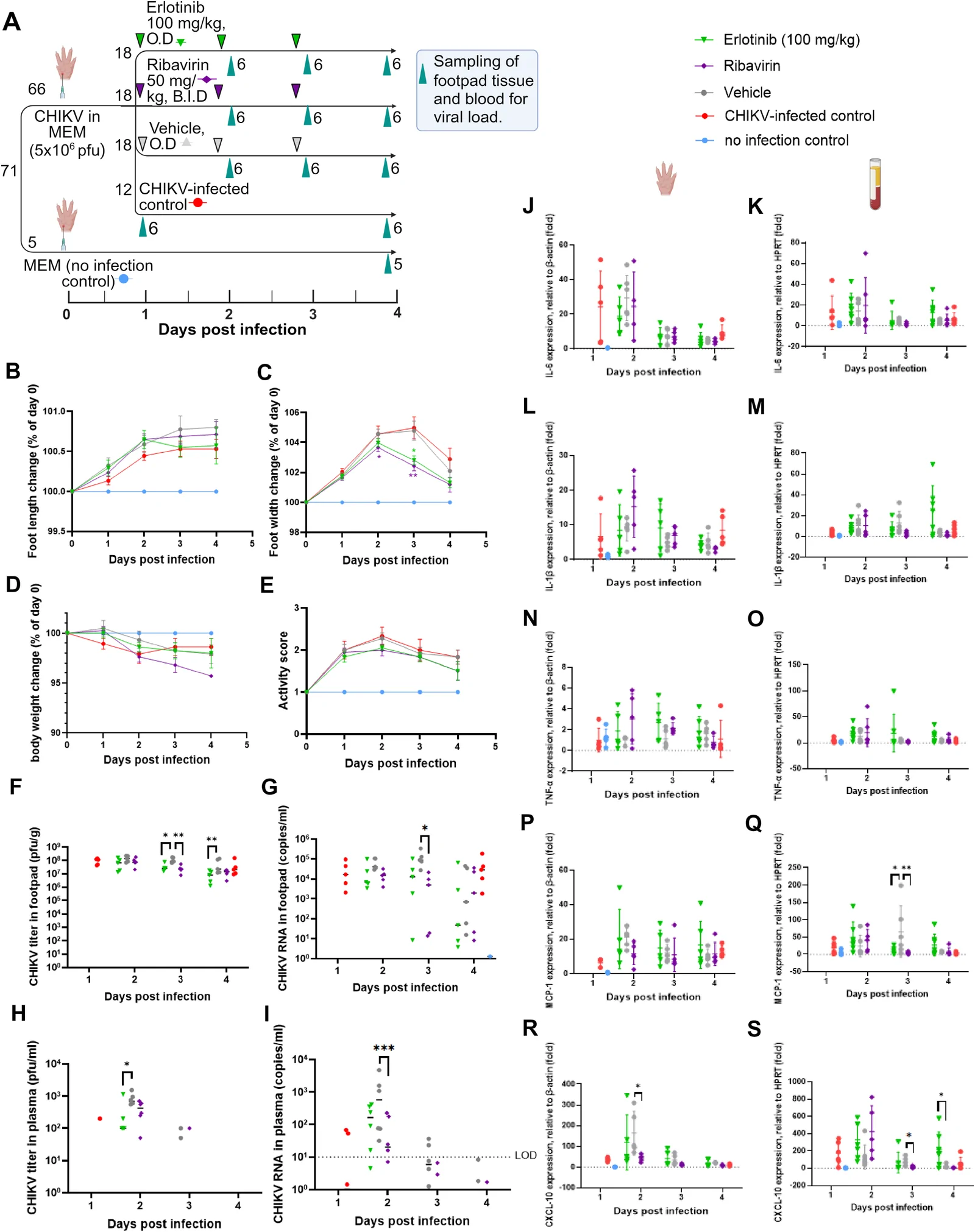
*In vivo* efficacy of erlotinib (100 mg/kg, once daily) in CHIKV-infected mice. (A) Schematic representation of the *in vivo* efficacy study design. C57BL/6 mice were inoculated subcutaneously in the footpad with CHIKV (5 x 10^6^ PFU) and treated with erlotinib (100 mg/kg, PO, once daily), ribavirin (50 mg/kg, IP, twice daily), or vehicle starting at 24 hours post-infection (hpi). (B–C) Clinical assessment of local inflammation measured by percentage change in footpad length and width relative to day 0. (D–E) Monitoring of systemic clinical parameters, including percentage body weight change and physical activity scores. (F–G) Quantification of viral burden in footpad tissues determined by plaque assay (PFU/g) and RT-qPCR (RNA copies/mL). **(H–I)** Systemic viral load in plasma measured by plaque assay (PFU/mL) and RT-qPCR (RNA copies/mL). (J–S) Relative mRNA expression levels of pro-inflammatory cytokines in footpad tissues (left column) and blood samples (right column) normalized to housekeeping genes β-actin and HPRT, respectively. Tissue cytokines include (J) IL-6, (L) IL-1 β, (N) TNF- α, (P) MCP-1, and (R) CXCL-10. Corresponding systemic cytokine profiles in blood include (K) IL-6, (M) IL-1β, (O) TNF-α, (Q) MCP-1, and (S) CXCL-10. Data is presented as mean ± SEM (n = 5-6 per group). Statistical significance of viral load and cytokine expression were determined by two-way ANOVA on log-transformed values followed by Dunnett’s multiple comparisons test comparing treatments to vehicle control at each time point (p<0.05). Raw fold change values / viral titers are displayed on the Y-axis for visualization. Statistical analysis of clinical parameters was evaluated with unpaired t-test (*p* < 0.05).

Among the clinical parameters (Fig 6B-E), assessment of footpad dimensions revealed modest clinical effects. Erlotinib and ribavirin treatment resulted in a transient significant reduction (*p* < 0.05, unpaired t-test) in foot width change by day 3 post-infection (Fig 6C). In contrast to the intensive QID regimen, the OD schedule was well-tolerated and did not result in drug-associated weight loss. Fig 6D–E shows that erlotinib-treated mice maintained a stable body weight percentage and physical activity scores throughout the 4-day observation period.

The antiviral evaluation of the OD regimen demonstrated selective, time-dependent efficacy *in vivo*. Despite the pharmacokinetics profile observed for once-daily dosing, erlotinib treatment significantly reduced footpad viral titers (PFU/g) (Fig 6F) on days 2 and 3 post-treatment, although footpad viral RNA levels remained largely unchanged (Fig 6G) compared to vehicle-treated controls. Systemic viral loads were only partially attenuated, evidenced by the significant reduction in plasma viral titers at day 1 post-treatment (Fig 6H) although plasma viral RNA levels (copies/mL) showed no significant difference (Fig 6I). At days 2 and 3 post-treatment, plasma viral titers and RNA levels fell below the limit of detection (LOD) across almost all experimental groups, resulting in floor-effect baseline values across cohorts at these time points. These findings indicate that the OD regimen achieved partial local and systemic viral suppression *in vivo* during acute infection.

Extensive analysis of the host inflammatory response showed that the OD regimen produced limited and variable immunomodulation, largely failing to reach statistical significance. In the infected footpad tissues, the expression levels of pro-inflammatory cytokines, including IL-6 (Fig 6J), IL-1β (Fig 6L), TNF-α (Fig 6N), MCP-1 (Fig 6P), and CXCL-10 (Fig 6R), were statistically indistinguishable from the vehicle-treated group across all time points. Similarly, systemic expressions of IL-6 (Fig 6K), IL-1β (Fig 6M), and TNF-α (Fig 6O) in blood samples remained comparable to controls. However, systemic chemokine profiling revealed variable effects: erlotinib treatment significantly suppressed blood MCP-1 expression at day 2 post-treatment (Fig 6Q), whereas CXCL-10 levels unexpectedly showed a statistically significant elevation at day 3 post-treatment (Fig 6S). Consequently, these findings indicate that while the OD regimen improves tolerability, it exerts partial antiviral effects providing only localized suppression with modest, chemokine-specific systemic modulation but does not achieve a broad or consistent suppression of CHIKV-induced cytokine production.

Histopathological analysis revealed that tissue inflammation decreased in the erlotinib- and ribavirin-treated groups, compared to the vehicle treatment (Fig 7). Consistent with this, blinded histopathologic scoring of representative footpad sections showed significantly reduced inflammation in erlotinib-treated mice relative to vehicle-treated controls. However, besides the transient significant reduction in foot width changes, no marked improvements were observed in clinical parameters, including body weight and activity scores, when compared with vehicle-treated mice. Overall, results support a modest host-directed effect.

**Fig 7:**
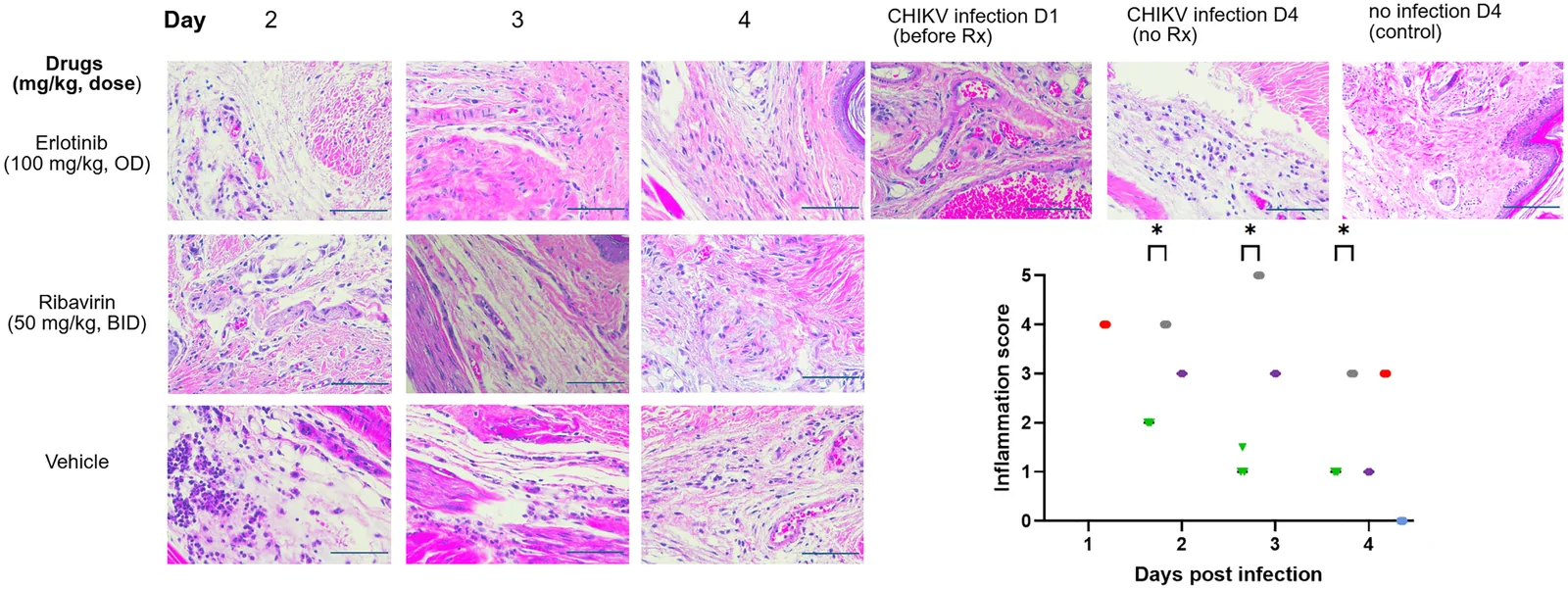
Histopathological analysis of footpad tissues of OD high-dose erlotinib in CHIKV-infected mice. Representative H&E-stained sections of footpad tissues. Controls include uninfected mice (Day 4) and CHIKV-infected mice prior to treatment (Day 1 post-infection). Treatment groups show tissue collected after 1, 2, and 3 days of administration with vehicle, erlotinib (100 mg/kg, OD), or ribavirin (50 mg/kg, IP, BID). Sections were assessed for leukocyte infiltration and structural changes. The scale bars represented 100 µm. Histological tissue inflammation was scored, and statistical analysis was performed using two-way ANOVA followed by Dunnett’s multiple comparisons test comparing treatments to vehicle control at each time point with significance set at * p < 0.05.

Altogether, these results demonstrate that, unlike the QID regimen, once daily oral administration of erlotinib exerts a measurable, targeted, and time-dependent, partial antiviral activity *in vivo*, reducing local footpad and systemic viral titers. Furthermore, its ability to selectively modulate systemic chemokines—evidenced by day 2 post-treatment MCP-1 suppression and the modulation of inflammation scores supports its potential role as a host-targeted therapeutic rather than a direct-acting antiviral.

## Discussion

This study validated the selection of erlotinib as a potential anti-CHIKV agent through *in vitro* screening, where it demonstrated potent antiviral activity and a favorable therapeutic window in Vero cells (Fig 1), consistent with its known broad-spectrum antiviral potential [11,12]. However, translating this efficacy to an *in vivo* murine model revealed significant pharmacokinetic (PK) constraints. While well-tolerated at a standard 30 mg/kg dose, erlotinib’s rapid clearance in mice (half-life of ~2–3 hours) failed to sustain therapeutic plasma levels over time (Fig 2L). To overcome this rapid murine clearance and establish a sustained drug level, we implemented an intensive four-times-daily (QID) high-dose (100 and 150 mg/kg) oral regimen. Results showed that the drug levels were consistently maintained above the *in vitro* EC_90_ threshold in the plasma (Fig 4F). While a 100 mg/kg QID schedule may appear excessive for clinical translation, it was a mechanical necessity in the murine model. In humans, erlotinib exhibits a substantially longer half-life (~36 hours) [33]; therefore, the effective dose for sustaining the drug level might be reduced in humans [34]. The 15% body weight loss seen in the QID group (Fig 4C, 4J) reflects a temporary physiological stress response to frequent administration rather than permanent organ toxicity, as it reverses once treatment stops [35,36]. In this study, the erlotinib also demonstrated a notable potential as a host-modulatory therapy aimed at mitigating inflammation and tissue pathology (Fig 5, 7). Despite failing to achieve virological control and inducing reversible physiological stress, the intensive QID regimen successfully preserved tissue architecture and reduced both edema and leukocyte infiltration. Conversely, the once-daily (OD) regimen yielded a significant antiviral activity (Fig 6F-H), reduced MCP-1 and increased CXCL-10 on day 2 and 3 post-treatment, respectively. Specifically, while continuous EGFR suppression during the QID regimen likely impaired essential homeostatic tissue repair processes, the intermittent inhibition achieved by the OD schedule struck a critical balance—restricting viral replication while preserving enough EGFR activity to facilitate host recovery.

Erlotinib is a known EGFR inhibitor. It plays a complex, context-dependent role during viral infection, functioning either as an enabler or suppressor of replication depending on the viral state and receptor activity [37]. Consequently, EGFR signaling requires tight regulation during infection: while overactive signaling can exacerbate viral pathogenesis, baseline EGFR activity remains essential for epithelial tissue repair. For instance, aberrant EGFR activation promotes pulmonary fibrosis and worsens lung pathology following severe acute respiratory syndrome coronavirus (SARS-CoV) infection, whereas therapeutic modulation of this pathway confers a protective effect [38]. Because EGFR governs critical homeostatic functions, its sustained inhibition presents an inherent trade-off between achieving antiviral efficacy and impairing the restoration of virus-damaged tissues [39]. In this context, the continuous EGFR suppression induced by the QID regimen likely over-suppressed these critical repair mechanisms. In contrast, the OD schedule achieved a balanced, intermittent inhibition that limited viral replication while preserving sufficient homeostatic signaling to facilitate tissue recovery. Additionally, the drug-induced weight loss—potentially linked to diarrhea-associated dehydration, may have indirectly weakened host defenses, further contributing to the lack of virological control observed in the QID-treated cohort [35,36,40,41].

In this study, the treatment was initiated at 24 hours post-infection, reflecting standard clinical delays [42]. Erlotinib was reported to inhibit host kinases, AAK1 and GAK, which regulate clathrin-mediated intracellular trafficking during entry and assembly/egress across multiple virus families [11,43,44]. In hepatitis C virus-infected cells, anti-attachment and time-of-addition studies with erlotinib and sunitinib have shown that these inhibitors act at a post-binding step in entry, indicating a dependence on the presence of the drug during an early window of infection [43]. Similarly, in a murine dengue model, the same drug combination provided protection against mortality even when treatment was initiated at multiple time points after infection, but this protection was not durable when treatment was delayed to 48 hours post-infection, with mice in this group eventually succumbing to infection [11]. These findings support AAK1/GAK as time-sensitive, host-directed antiviral targets in other viral systems but do not establish that erlotinib acts similarly against CHIKV, since the present study did not test treatment timing or perform entry-, attachment-, or target-specific mechanistic assays. The limited *in viv*o efficacy observed following administration at 24 hours post-infection may simply reflect delayed treatment timing relative to the viral life cycle, rather than an inherent failure to block viral entry. Comparative studies using different treatment schedules and stage-specific assays will elucidate these discrepancies. Whether AAK1/GAK inhibition drives anti-CHIKV activity also remains to be directly established, and resolving this mechanistic question is essential for advancing erlotinib toward further development.

The immunomodulatory profile of the OD regimen further underscores the host-directed mechanism of erlotinib. The observed reduction in serum MCP-1 (Fig 6Q) supports the mitigation of inflammation, as MCP-1 is a well-established driver of CHIKV joint pathology and its pharmacological inhibition reduces disease severity in CHIKV mouse models [45,46]. In our study, this MCP-1 reduction coincided with decreased viral titers and histopathologic inflammation, although direct causality was not tested. However, this potential clinical benefit may be offset by the concurrent elevation of CXCL-10 (Fig 6S), an interferon-stimulated chemokine known to drive macrophage-mediated joint inflammation and potentially exacerbate local pathology [47,48]. Overall, the partial antiviral activity and mixed chemokine responses observed under the OD regimen indicated that erlotinib's net effect on host immunity during CHIKV infection remains incompletely understood and warrants further investigation.

Furthermore, the choice of oral (PO) administration likely contributed to the *in vivo* efficacy gap observed here compared to other viral models. Erlotinib undergoes extensive first-pass hepatic metabolism following oral administration [49,50]. Since all *in vivo* efficacy assessments in this study utilized this route, we cannot rule out route-dependent limitation in bioavailability. In prior studies of dengue and Ebola virus infections, intraperitoneally administered erlotinib alone did not significantly reduce viremia or improve survival. Therapeutic benefits were only achieved when combined with sunitinib, and oral administration was evaluated exclusively for this combination [11]. This suggested that erlotinib's *in vivo* antiviral activity may be inherently modest as a monotherapy, regardless of the administration route, and that our findings are broadly consistent with this established pattern. Notably, while the prior study achieved efficacy using comparatively moderate intraperitoneal doses (30–60 mg/kg), our oral QID regimen required considerably higher cumulative dosing to sustain adequate plasma exposure. Bypassing first-pass hepatic metabolism [51] could potentially achieve therapeutic concentrations at the lower, better-tolerated dose used in our OD regimen, which might improve upon the modest antiviral activity observed. Ultimately, oral erlotinib may be more appropriately positioned as an adjunctive immunomodulatory therapy alongside direct-acting antivirals, rather than a standalone treatment.

Finally, the validated *in vitro* activity underscores the quinazoline core as a highly viable antiviral scaffold. Future efforts should prioritize Structure-Activity Relationship (SAR) studies. Developing quinazoline derivatives with structural optimizations is required to retain the host-kinase targeted antiviral efficacy while securing enhanced metabolic stability and PK profiles suitable for *in vivo* success [52,53].

In conclusion, while erlotinib exhibits clear anti-CHIKV potential *in vitro*, its standalone *in vivo* antiviral efficacy showed a dosing regimen-dependent activity – with partial efficacy observed under the once-daily oral dosing regimen. The partial *in vivo* efficacy may, in part, be related to its rapid clearance and transient exposure relative to the concentrations associated with antiviral activity *in vitro*; whether parenteral administration, which bypasses first-pass hepatic metabolism, would improve systemic exposure and efficacy remains to be determined. The precise mechanism and stage of the CHIKV replication cycle targeted by erlotinib also remain unresolved and warrant further investigation. Future development should explore prophylactic timing, parenteral routes of administration, synergistic antiviral combinations, and SAR-guided structural optimization.

## Supporting information

S1 Fig LC-MS/MS method validation for erlotinib quantification. (A) Product ion mass spectrum showing the transition from the precursor ion (m/z 393.900) to the product ion (m/z 278.000) used for erlotinib quantification. (B) Calibration curve for erlotinib quantification by LC-MS/MS, generated over a concentration range of 0.07–3,500 ng/mL (Y = 15,293.6x + 2,554.81; R² = 0.99920).
(DOCX)

S1 Table Bioanalytical validation and pharmacokinetic sampling design for erlotinib quantification. (A) Back-calculated concentrations, percent accuracy, and calibration curve parameters (Y = 15,293.6x + 2,554.81; R² = 0.99320) for erlotinib in blank mouse plasma at nominal concentrations (CC1–CC8) ranging from 0.070–3,500 ng/mL. (B) Alternating sparse-sampling scheme, showing blood collection time points (hours/days) assigned to each of four mouse groups.
(DOCX)

S2 Table List of mouse primers for cytokines. (DOCX)

S2 Fig H & E pathology of liver, kidney and spleen samples on day 1, 3, and 7 after administering a single dose of 30 mg/kg of erlotinib. The line represents a 100 µm scale.
(DOCX)

S3 Fig H & E pathology of liver, kidney and spleen samples on day 4 following a repeated oral dose administration of erlotinib (30 mg / kg) for 3 days. The line represents a 100 µm scale.
(DOCX)

S4 Fig Chromatograms of erlotinib (at 750 ng/mL) into blank mouse plasma. (DOCX)

S5 Fig H & E pathology of liver, kidney and spleen samples on day 4 following a multiple-dose administration of erlotinib either orally (100, 150 mg / kg) or intraperitoneally (100 mg / kg) for 3 days. The line represents a 100 µm scale.
(DOCX)

S6 Fig H & E pathology of liver, kidney and spleen samples on day 4 following multiple-dose administration of erlotinib (100 and 150 mg / kg) given orally four times daily for 3 days. The line represents a 100 µm scale.
(DOCX)
