# Supplementary material for: Erlotinib in a once-daily high-dose oral regimen could mitigate Chikungunya virus titer in mice
Source: PLoS One. 2026 Nov 2;21(11):e0360169. doi: 10.1371/journal.pone.0360169 (PMC13726984; doi:10.1371/journal.pone.0360169)
Supplement: Supplementary file 1 [file pone.0360169.s001.docx]

**S1 Fig**

**A**

**LC-MS/MS spectra of erlotinib (393.900→278.00)**

**B**

**Calibration curve of erlotinib at 0.07- 3,500 ng/mL**
