# Supplementary material for: Erlotinib in a once-daily high-dose oral regimen could mitigate Chikungunya virus titer in mice
Source: PLoS One. 2026 Nov 2;21(11):e0360169. doi: 10.1371/journal.pone.0360169 (PMC13726984; doi:10.1371/journal.pone.0360169)
Supplement: Supplementary file 2 [file pone.0360169.s002.docx]

**S1 Table**

**A**

| **Parameter** | **Back-calculated concentrations of erlotinib in mice plasma (ng/ml)** | | | | | | | | **Equation of**  **calibration curve** | **R^2^** |
| --- | --- | --- | --- | --- | --- | --- | --- | --- | --- | --- |
|  | **CC1** | **CC2** | **CC3** | **CC4** | **CC5** | **CC6** | **CC7** | **CC8** |  |  |
| Back cal. | 0.071 | 0.296 | 1.642 | 6.713 | 37.329 | 171.409 | 724.709 | 3258.571 | Y = (15293.6)X + (2554.81) | 0.99320 |
| % Accuracy | 101.429 | 89.697 | 109.394 | 93.016 | 109.649 | 106.740 | 96.576 | 93.102 |  |  |
| Nominal value (ng/ml) | 0.070 | 0.330 | 1.501 | 7.217 | 34.044 | 160.586 | 750.400 | 3,500.000 | - | - |

**B**

| **Alternating group** | **Time-points for blood draw** |
| --- | --- |
| 1 | 1, 5, |
| 2 | 2, 6, |
| 3 | 3, 12, |
| 4 | 0.5, 4, 24 |
