# Supplementary material for: Erlotinib in a once-daily high-dose oral regimen could mitigate Chikungunya virus titer in mice
Source: PLoS One. 2026 Nov 2;21(11):e0360169. doi: 10.1371/journal.pone.0360169 (PMC13726984; doi:10.1371/journal.pone.0360169)
Supplement: Supplementary file 3 [file pone.0360169.s003.docx]

**S2 Table**

**List of mouse primers for cytokines**

| **Gene** | **Sequence** |
| --- | --- |
| IL-6 | For: 5’-GACAAAGCCAGAGTCCTTCAGA-3’  Rev: 5’-TGTGACTCCAGCTTATCTCTTGG-3’ |
| IL-1b | For: 5’-GCCACCTTTTGACAGTGATGAG-3’  Rev: 5’-GACAGCCCAGGTCAAAGGTT-3’ |
| TNF-α | For: 5’ -CGAGTGACAAGCCTGTAGCC-3’  Rev: 5’-ACAAGGTACAACCCATCGGC-3’ |
| MCP-1 | For: 5’-CAGGTCCCTGTCATGCTTCT-3’  Rev: 5’-GAGTGGGGCGTTAACTGCAT-3’ |
| CXCL10 | For: 5’-GGTCTGAGTCCTCGCTCAAG-3’  Rev: 5’-GTCGCACCTCCACATAGCTT-3’ |
| β-actin | For: 5’-CGG TTC CGA TGC CCT GAG GCT CTT–3’  Rev: 5’-CGT CAC ACT TCA TGA TGG AAT TGA–3’ |
| Hprt-1 | For: 5’-GCAGCGTTTCTGAGCCATTG-3’  Rev: 5’-TCATCGCTAATCACGACGCT-3’ |
