# Supplementary material for: Erlotinib in a once-daily high-dose oral regimen could mitigate Chikungunya virus titer in mice
Source: PLoS One. 2026 Nov 2;21(11):e0360169. doi: 10.1371/journal.pone.0360169 (PMC13726984; doi:10.1371/journal.pone.0360169)
Supplement: Supplementary file 4 [file pone.0360169.s004.docx]

**S2 Fig**

**H & E pathology of liver, kidney and spleen samples on day 1, 3, and 7 after administering a single dose of 30 mg/kg of erlotinib.** The line represents a 100 µm scale.
