# Supplementary material for: Erlotinib in a once-daily high-dose oral regimen could mitigate Chikungunya virus titer in mice
Source: PLoS One. 2026 Nov 2;21(11):e0360169. doi: 10.1371/journal.pone.0360169 (PMC13726984; doi:10.1371/journal.pone.0360169)
Supplement: Supplementary file 5 [file pone.0360169.s005.docx]

**S3 Fig**

**H & E pathology of liver, kidney and spleen samples on day 4 following a repeated oral dose administration of erlotinib (30 mg / kg) for 3 days.** The line represents a 100 µm scale.
