# Supplementary material for: Erlotinib in a once-daily high-dose oral regimen could mitigate Chikungunya virus titer in mice
Source: PLoS One. 2026 Nov 2;21(11):e0360169. doi: 10.1371/journal.pone.0360169 (PMC13726984; doi:10.1371/journal.pone.0360169)
Supplement: Supplementary file 6 [file pone.0360169.s006.docx]

**S4 Fig**

**Chromatograms of erlotinib (at 750 ng/mL) into blank mouse plasma.**
